# Violet bioluminescent *Polycirrus* sp. (Annelida: Terebelliformia) discovered in the shallow coastal waters of the Noto Peninsula in Japan

**DOI:** 10.1038/s41598-021-98105-6

**Published:** 2021-09-27

**Authors:** Shusei Kanie, Daisuke Miura, Naoto Jimi, Taro Hayashi, Koji Nakamura, Masahiko Sakata, Katsunori Ogoh, Yoshihiro Ohmiya, Yasuo Mitani

**Affiliations:** 1 Bioproduction Research Institute, National Institute of Advanced Industrial Science and Technology (AIST), Sapporo, 062-8517 Japan; 2 Biomedical Research Institute, AIST, Tsukuba, 305-8566 Japan; 3 National Institute of Polar Research, Tachikawa, Tokyo 190-8518 Japan; 4 Olympus Corporation, Hachioji, Tokyo 192-8512 Japan; 5 Japan Underwater Films Co., Ltd., 2-11-15, Nakaochiai, Shinjyuku, Tokyo 161-0032 Japan; 6 Biomedical Research Institute, AIST, Ikeda, 563-8577 Japan; 7 Osaka Institute of Technology (OIT), Osaka, 535-8585 Japan; 8 Present Address: Sugashima Marine Biological Laboratory, Graduate School of Science, Nagoya University, Toba, Mie, 517-0004 Japan; 9 HATENOURUMA, Hachioji, Tokyo, 192‑0023 Japan

**Keywords:** Marine biology, Biooceanography

## Abstract

Terebellidae worms have large numbers of tentacles responsible for various biological functions. Some Terebellidae worms whose tentacles emit light are found around the world, including exceptional violet-light-emitting *Polycirrus* spp. found in Europe and North America. However, there is no video-recorded observation of the luminous behavior of such unique species in nature, and the genetic information related to their ecology are lacking. Here, for the first time, we video-recorded the violet-light-emitting behavior of an undescribed Japanese worm in its natural habitat. The worm was designated as *Polycirrus* sp. ISK based on morphological observations, and the luminescence spectrum showed a peak at 444 nm, which is an exceptionally short wavelength for bioluminescence in a shallow coastal water environment. An analysis of differentially expressing genes based on separate RNA-Seq analysis for the tentacles and the rest of body revealed the specific expression of genes that are probably involved in innate immunity in the tentacles exposed to predators. We also found a *Renilla* luciferase homologous gene, but coelenterazine was not detected in the worm extract by analyses using a liquid chromatography and a recombinant *Renilla* luciferase. These results will promote an understanding of the ecology and luminescence mechanisms of luminous *Polycirrus* spp.

## Introduction

Luminous animals are diverse, and more than 700 genera have been found to contain light-emitting species^1,2^. The color of the emitted light depends on the species, and its biological meaning has been discussed from various aspects, such as habitat or luminous behavior^3–5^. One of the notable observations is the relationship between habitat depth and color of bioluminescence in the ocean^6^. In marine luminous animals, the majority of deep-sea species emit blue light with wavelengths around 475 nm, and green light with wavelengths around 500 nm is the most common color in shallow-water species^6–8^. Exceptionally few species produce violet light having wavelengths shorter than 450 nm^8^. Light absorption and scattering by seawater would explain the biological significance of blue and green bioluminescence depending on habitat depth^6^, whereas the function of violet bioluminescence is still obscure due to the small number of reports of violet-light-emitting species.

Polychaetes, which are mostly marine species, include eight families of luminous species: Acrocirridae, Chaetopteridae, Cirratulidae, Flabelligeridae, Polynoidae, Syllidae, Tomopteridae, and Terebellidae^5^. The family Terebellidae includes unique species that emit violet light*.* Terebellidae species exhibit a characteristic morphological feature of conspicuous tentacles with important biological functions, including food acquisition, swimming ability, and a defense against predators ^9–11^. Despite these observations for the tentacle’s function related to the ecology, few molecular-level studies of the related families have been performed^12,13^. Among Terebellidae, luminous species are found in two genera: *Thelepus* and *Polycirrus*. The previous chemical study on metabolites from *Thelepus* spp. revealed an antimicrobial compound localized in their tentacles^14^. Recently, the Japanese *Thelepus japonicas*, which emits light at λ_max_ 508 nm, was studied with a focus on the molecular mechanism underlying light emission^15^, while the molecular bases of luminous *Polycirrus* spp. remain to be clarified.

Luminous *Polycirrus* spp. have long been known in various places around the world^16,17^, including *Polycirrus auranticus* from the coast of England^9^ and *Polycirrus preplexus* from California^17^. The former species is reported to show a rather weak violet–blue light flashing out at the tips of tentacles when the worm is disturbed^9^. The latter is reported to be a nonsecretion flash-type light emission, and an analysis using a charge-coupled device (CCD) spectrophotometer revealed that the emitting light had a 445 nm emission peak^17^. However, almost all descriptions of luminous *Polycirrus* spp. in the literature are more than 30 years old and lack clear photographs or videos that would suggest these species’ ecological behaviors.

In this study, for the first time, we video-recorded the violet-light-emitting behavior of an undescribed worm in the shallow coastal waters of the Noto Peninsula, Ishikawa, Japan. The worm was morphologically identified and named *Polycirrus* sp. ISK. In addition, we successfully collected the light-emission spectrum with a peak at 444 nm, which was very similar to that of *P. preplexus* found in California. Our RNA-Seq analysis showed that the existence of a gene coding for fucolectin, which is a fucose-binding lectin related to an innate immunity response, was significantly enriched in the tentacles. The RNA-Seq data included a homologous gene of *Renilla* luciferase, which is the enzyme responsible for coelenterazine-dependent bioluminescence, but coelenterazine was not detected in the worm extract by analyses using ultra performance liquid chromatography (UPLC) and a recombinant *Renilla* luciferase.

## Results and discussion

### Morphology and light-emitting behavior of the undescribed Japanese Terebellidae worm

**Figure 1:**
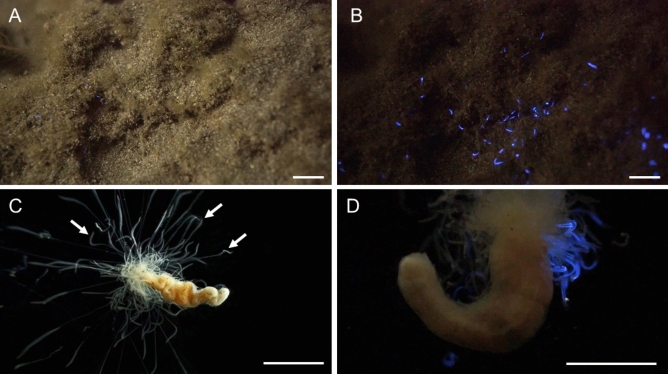
Photographs of *Polycirrus* sp. ISK. (**A**) *Polycirrus* sp. ISK in its natural habitat with bright-field illumination. (**B**) Bioluminescence of *Polycirrus* sp. ISK in its natural habitat without bright-field illumination. The worms were stimulated by air bubbling from SCUBA gear. (**C**) A single worm with stretched tentacles. Tentacles are indicated by white arrows. (**D**) The worm with light emission at the tentacles. This worm was stimulated by an electric shock. Scale bars = 100 mm for A and B, 10 mm for C and D. Each photograph was extracted from the videos recorded with the following settings: sensitivity, ISO 51200 or 11 lx; white balance, 4300 K or 5800 K; shutter speed, 1/30 s or 1/60 s; iris, F1.8-3.5; frame rate, 29.97 fps or 60 i; frame size, 1920 × 1080 pixels. Original high quality videos are available at https://youtu.be/KEsU0kWAEfg and https://youtu.be/24dxvPlBDB0

**Figure 2:**
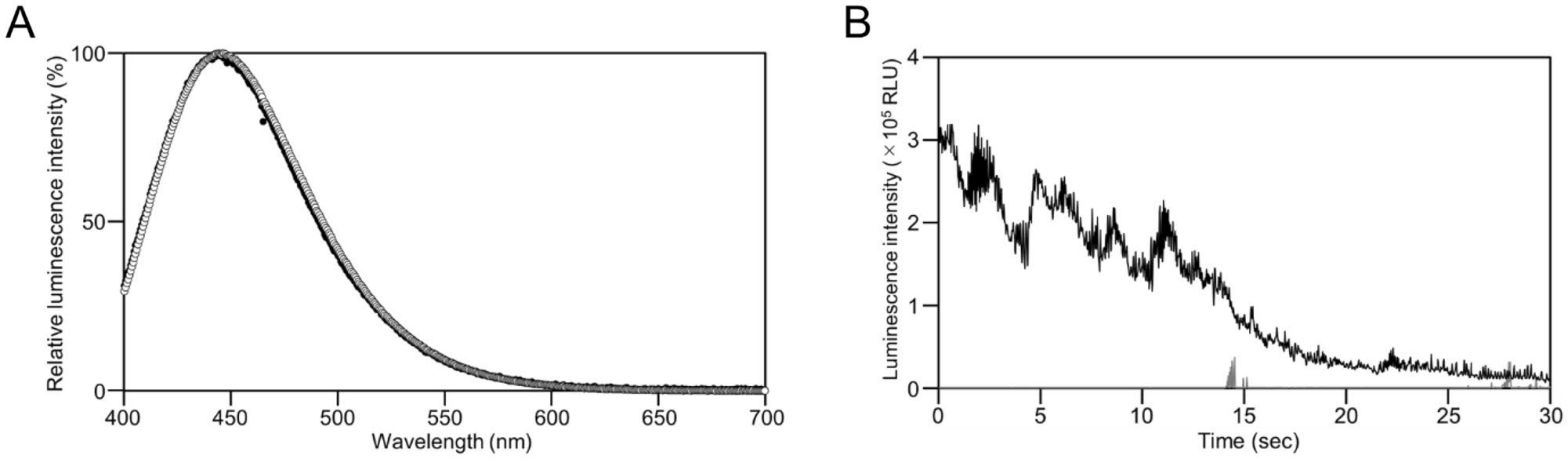
Luminescence spectra and KCl-induced light emission of *Polycirrus* sp. ISK. (**A**) Spectrum analysis of *Polycirrus* sp. ISK using a living worm stimulated by an electric shock. The luminescence spectra were obtained from two different individuals. The λ_max_ represented in closed circles and open circles were 444 nm and 446 nm, respectively. (**B**) Typical light-emission signal of a living worm soaked in 667 mM KCl. The black line indicates luminescence intensity after adding KCl solution, and the gray line indicates luminescence intensity before adding KCl solution.

In 2016, some of the present authors were exploring shallow coastal waters (depth less than 1 m) to observe the ecological behaviors of marine animals in the Noto Peninsula, when they discovered unknown violet-light-emitting worms. At the sampling point, the worms were living in small holes (a few centimeters in diameter) or in cracks in rocks covered by sand at the shallow sea bottom (Supplementary Fig. S1). We successfully video-recorded their emission of violet light from the whole tentacle stretching into sea water when stimulated by air bubbling at night (Fig. 1A–C; Supplementary Videos S1 and S2). The violet-light emission consisted of rapid flashes with variable duration in the order of milliseconds (Supplementary Video S3), as observed for the worm *P. perplexus* in response to stimulation^17^. From our morphological observation, we identified the violet-light-emitting worm as a member of *Polycirrus* on the basis of the following characteristics^18^: (1) a sheetlike prostomium covering the upper lip; (2) avicular unicini on some neuropodia; (3) no branchiae. The specimens also have the following characteristics: (1) neurochaetae beginning on last notochaetigerous segment, chaetiger 14; (2) uncini with a long neck and concave base; (3) notopodial pre- and post-chaetal lobes both similar shape. These characters are also found in *Polycirrus disjunctus* Hutchings and Glasby^18^; however some of the characters in parapodial lobes and chaetae have differentiation. Thus, we concluded that this species should be treated as an undescribed species. Further comparative observation is needed to describe the species. At this time we treated the *Polycirrus* species observed in this study as *Polycirrus* sp. ISK. Application of an electric pulse also caused clear light emission from the tentacles of the living worm (Fig. 1D; Supplementary Video S3), and the luminescence spectrum showed that its λ_max_ was 444 nm or slightly longer, depending on the individual (Fig. 2A). We also found that light emission was efficiently induced by the addition of KCl solution and observed the time course of light emission with rapid fluctuations with variable duration in the order of milliseconds for up to 30 s (Fig. 2B). The flash pattern was similar to that observed in a study of *P. perplexus*^17^. In the genus *Polycirrus*, *P. medius* and *P. nervosus* in Japan have been described^18,19^. However, the morphological features of the species in the present study differed from these species on the basis of our observations described above.

Japanese *Polycirrus* spp. have not been described as luminous worms according to our review of the literature and web pages. In addition, the number of reports for new *Polycirrus* spp. from all over the world has been increasing, but a limited number of species are known to emit light^13,17,18^. Our finding of KCl-induced light emission from *Polycirrus* sp. ISK suggested that we can easily test the light-emitting ability of *Polycirrus* spp. by luminescence measurement just after adding KCl solution. A spectrum pattern has been reported for only one species, *P. perplexus* collected in California^17^, and it would be necessary for further understanding of these species to examine the light-emitting abilities and to compare light-emitting behaviors and spectrum patterns. The color of bioluminescence is often related to habitat, and light in the blue range is typical for pelagic species^20^. Thus, one of the points to be focused on is the ecological function of the violet-light emission of this worm inhabiting in a shallow coastal water environment. In *P. perplexus*, deterring predation is a possible function of luminescence based on that species’ habitat and its violet-light emission^17,21^. As shown in Supplementary Videos S1, S2, which are the first video records of in situ light emission of a *Polycirrus* species, the air bubble-stimulated luminescence of *Polycirrus* sp. ISK in its natural habitat also seemed to deter predation, but this explanation is still speculative.

### Differentially expressing genes between the tentacles and the rest of body

A few years after discovering this worm, we found it difficult to collect enough of them to conduct common biochemical and chemical analyses because we did not find a place densely inhabited by hundreds of the worms whose wet weight was a few tens of milligrams (e.g. 16.5, 29.8, or 31.8 mg). Next, we conducted RNA-Seq analysis. In luminous animals with strong light emission, such as firefly or syllid polychaetes (Syllidae), luciferase expression is high especially at the luminous organ or in the whole body^22,23^. On the other hand, the light emission of *Polycirrus* sp. ISK was not so strong compared to that of fireflies, and the light-emitting area was limited to the tentacles. In addition, the genetic information related to the tentacles responsible for various ecological functions is still limited. Thus, in the present study we decided to purify RNA from the tentacles and the rest of body separately (Fig. 1C) and performed RNA-Seq analysis followed by a computational analysis using the MASER pipeline^24^. By de novo assembly, 110,775 contigs were predicted; 26.1% of them showed more than twice the expression level in the tentacles than in the rest of body, whereas 20.8% showed more than twice the expression in the rest of body than in the tentacles. When we performed a blastX search to the NCBI nr database for the contigs longer than 300 bp, 35.6% showed significant homology with registered genes with e-values of less than 1e^−10^. The average length for these contigs was 1384 bp, and half of them were in the range of 463–1863 bp (Supplementary Fig. S2). In the assembled sequence, we found the cytochrome oxidase subunit I (COI) gene and tried to construct a phylogenetic tree. However, the obtained phylogenetic tree was unreliable due to the low bootstrap values as shown in Supplementary Fig. S3.

To focus on the tissue-specific genes, we first picked up genes with high expression levels based on high fpkm values (over 1000) and then ranked these genes based on the tissue-specificity judged by the comparison of fpkm values in tentacles and the rest of body. In tentacle-specific genes, we found that some genes coding for lectin(-like) domains were ranked in the top eight as shown in Supplementary Table S1. Of the top eight genes in the rest of body-specific genes (Supplementary Table S2), seven exhibited no similarity to any genes, and the remaining gene exhibited significant similarity to a hypothetical protein of *Capitella teleta*, which is a Polychaetes species with whole-genome information available^25^. Recently, TPM is preferably used to normalize expression level, and the value is used for statistical differential expression analysis^26^, and we also calculated TPM for tissue-specific genes (Supplementary Table S3).

**Figure 3:**
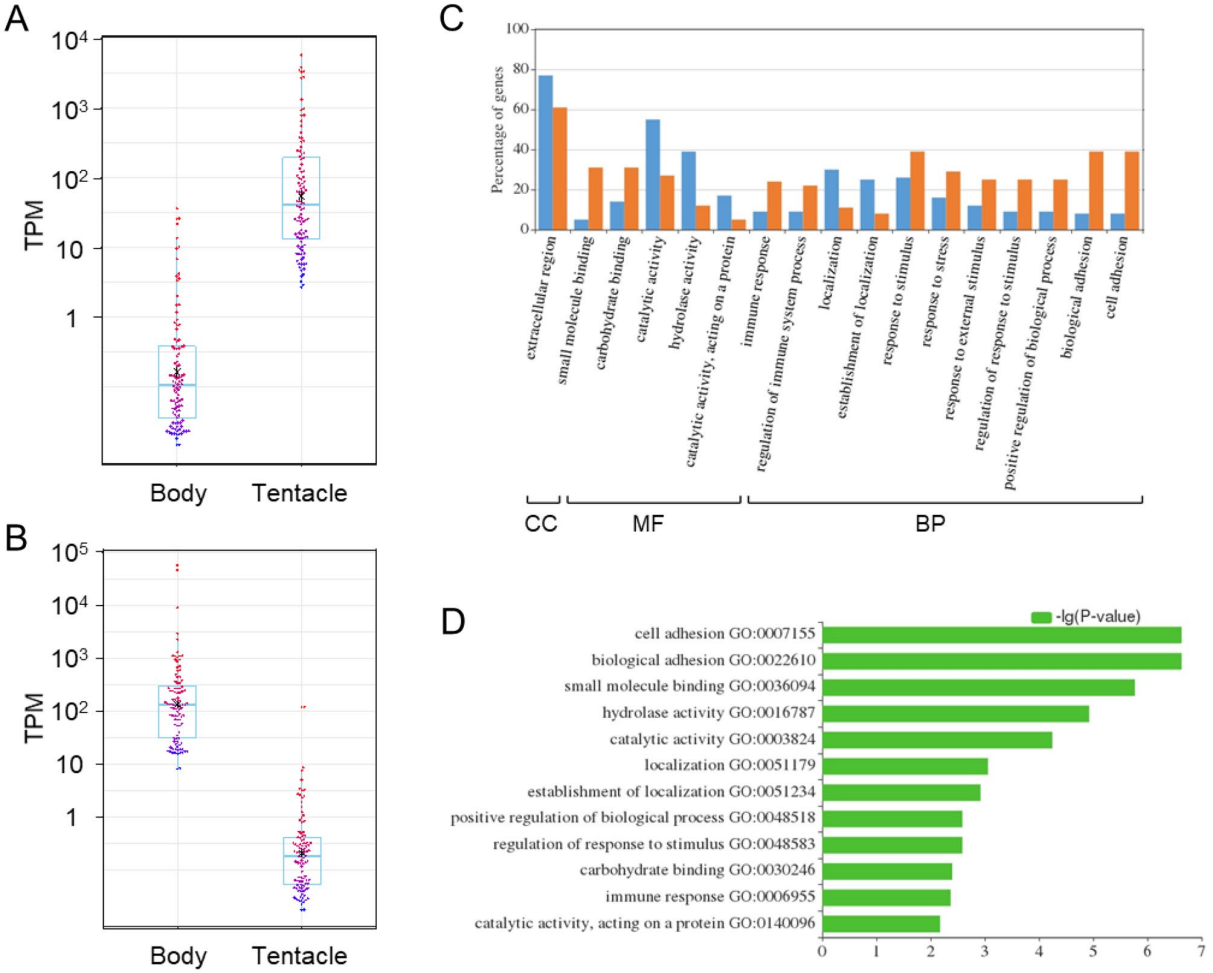
WEGO analysis of highly expressing genes in the tentacle and the rest of body. (**A**) Box plot graph for the distribution of TPM value for top 100 genes highly expressing in the tentacle. Corresponding genes in each part are colored in the same gradation color according to the TPM value (red to blue form higher to lower value). (**B**) Box plot graph for the distribution of TPM value for top 100 genes highly expressing in the rest of body. Each gene is colored as in (**A**). (**C**) WEGO analysis of top 100 genes highly expressing in the tentacle (orange bar) and the rest of the body (blue bar). (**D**) P-values from Chi-square tests obtained by WEGO analysis. *CC* cellular component, *MF* molecular function, *BP* biological process.

As we were unable to conduct statistical differential expression analysis due to no biological/technical replication resulted from difficulties in the sample collection, we simply compared TPM value between the tentacle and the rest of body samples. The ratio of TPM (tentacle/rest of body) was calculated, and then top 100 genes (Fig. 3A), which were highly expressing in the tentacle, were selected. Similarly, top 100 genes highly expressing in the rest of body were selected using the ratio of TPM (rest of body/tentacle) (Fig. 3B). These gene lists were annotated by gene ontology (GO) terms and analyzed using WEGO program^27^. WEGO results showed different expression patterns for the tentacle and the rest of body. In the tentacle, GO terms including cell adhesion, biological adhesion, small molecular binding, positive regulation of biological process, regulation of response to stimulus, carbohydrate binding, and immune response were significantly higher (Fig. 3C, D). In the rest of body, GO terms including hydrolase activity, catalytic activity, localization, and establishment of localization were significantly higher. In the top 100 genes highly expressing in the tentacle, we found 21 genes annotated as a gene coding for fucolectin by blast search (Supplementary Table S4). Fucolectin is a fucose-binding lectin involved in the innate immunity of diverse invertebrate species^28^. However, its function in invertebrates remains unclear, and no information is available for Terebellidae, including sequence information. Fucolectin was first identified in eel with mRNA distribution mainly in liver and gill^28^. In sea cucumber, expression of the fucolectin gene is confirmed in respiratory trees, muscle, and tentacle^29^. We were not able to see whether this gene was expressed in the respiratory organ of *Polycirrus* sp. ISK because a characteristic of the genus *Polycirrus* is the absence of branchiae^18^. Nevertheless, the tentacle-specific expression of fucolectin was consistent with the observation in sea cucumber, and the high expression of such proteins involved in innate immunity seemed reasonable because tentacles stretching out of their bodies can be damaged by attack of predators and thus are threatened by infectious bacteria and other pathogens^11^, as is the respiratory organ. In addition, localization of antimicrobial compounds in Terebellidae worms is suggested to be of antiseptic importance in damage by predation^14^. This study would provide indispensable information about the ecological meaning of *Polycirrus* sp. ISK’s life in future genetic studies.

### Transcripts coding for luciferase-like genes in the worm

To find genes similar to the known luciferase, which is an enzyme oxidizing a specific compound called luciferin to emit light, from related species in polychaetes, we performed a blastX analysis against the *Odontosyllis* luciferase sequence using our RNA-Seq data. We found a gene coding for a protein that exhibited similarity to *Odontosyllis* luciferase, but the e-value was more than 1e^−10^ (Supplementary Fig. S4). In addition, the top hit for this gene analyzed by blastX was annotated to code an uncharacterized protein of *Saccoglossus kowalevskii* (Hemichordata), and its specific function was not predicted. Other hits were for genes from Chordata, Mollusca, and other phyla but there was no hit from Annelida. This result would suggest that the light-emission system of *Polycirrus* sp. ISK differs from that of the genus *Odontosyllis*, although further experiments using high purity *Odontosyllis* luciferase and the substrate will be necessary to confirm this. In further blastX analyses of representative luciferases, photoproteins, and a putative luciferase [luciferases from the ostracod *Cypridina noctiluca* (Accession number: BAD08210.1), the copepod *Gaussia princeps* (AAG54095.1), the deep-sea shrimp *Oplophorus gracilirostris* (BAB13775.1 and BAB13776.1), the firefly *Photinus pyralis* (AAA29795.1), the sea pansy *Renilla reniformis* (AAA29804.1); photoproteins from the hydrozoan jellyfish *Aequorea victoria* (AAA27720.1), the hydroid *Clytia gregaria* (CAA49754.1), the hydroid *Obelia geniculate* (AAL86372.1); a putative luciferase from the tunicate *Pyrosoma atlanticum*^30^ sequences using our RNA-Seq data], we found some tissue-nonspecific genes whose sequences exhibited similarity to firefly luciferase (FLuc) or *Renilla* luciferase-like protein (RLuc-like) sequences with an e-values of less than 1e^−10^ and percent identity of more than 50%. FLuc is a member of the acyl-adenylate-forming superfamily of enzymes responsible for firefly luciferin-dependent bioluminescence, which is found in terrestrial luminous beetles emitting light ranging from green to red^31^. Previously, a putative acyl-CoA synthetase protein was found in the luminous organ of firefly squid emitting blue light^32^, but there is no clear biochemical evidence that such protein is responsible for firefly squid’s bioluminescence. On the other hand, RLuc is responsible for coelenterazine-dependent bioluminescence, which is found in marine luminous organisms belonging to various taxa. An RLuc-like protein is found to be localized in luminous organs of the brittle star *Amphiura filiformis*, as revealed by taking advantage of the cross reactivity of anti-RLuc antibody to *A. filiformis* RLuc-like protein^33^. A recent study reported that recombinant RLuc-like protein found in *P. atlanticum* exhibited luciferase activity to coelenterazine^30^. However, an RLuc-like protein from sea urchin *Strongylocentroutus purpuratus* is confirmed to exhibit dehalogenase activity to various substrates but no luciferase activity to coelenterazine^34^. Therefore, it is suspected that *Polycirrus* sp. ISK possesses a luminescence system using an RLuc-like enzyme.

### Coelenterazine content in the worm

**Figure 4:**
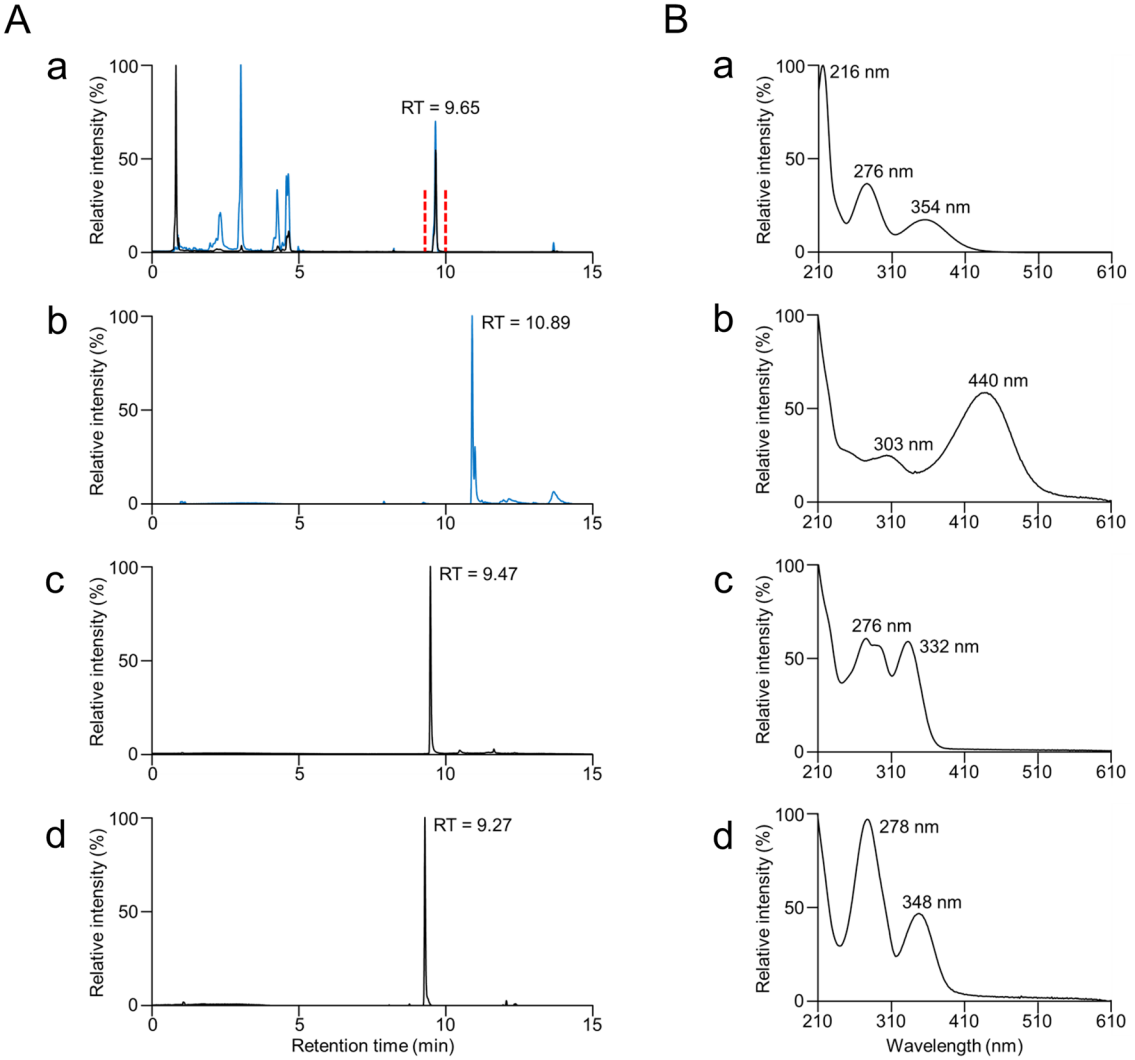
Comparison of the ethanolic extract of *Polycirrus* sp. ISK with CTZ, CTMD, and CTM. (**A**) UPLC analysis of (a) the extract, (b) authentic CTZ, (c) authentic CTMD, and (d) authentic CTM using a multiwavelength detector. The black solid line indicates detection at 333 nm, and the blue solid line indicates detection at 435 nm. The compound between the red vertical dashed lines was collected for MS/MS analysis. (**B**) Absorption spectra of the compound from the extract, CTZ, CTMD, and CTM obtained at retention times of (a) 9.65, (b) 10.89, (c) 9.47, and (d) 9.27 shown in (**A**). *CTZ* coelenterazine, *CTMD* coelenteramide, *CTM* coelenteramine. These chemical structures are shown in Supplementary Fig. S5.

To investigate whether *Polycirrus* sp. ISK possesses not only a *Renilla* luciferase homologous gene but also coelenterazine, we analyzed an ethanolic extract of *Polycirrus* sp. ISK by UPLC with a UV–visible detector (Fig. 4). The obtained UPLC chromatogram did not show a peak corresponding to that of authentic coelenterazine. When further checking the chromatogram, we found the peak at a retention time similar to those of authentic coelenteramide and coelenteramine, which can be formed from coelenterazine. However, the absorption spectrum obtained by UPLC analysis and the mass spectrum obtained by MS/MS analysis were not identical to those of authentic coelenteramide or coelenteramine (Fig. 4 and Supplementary Figs. S5 and S6). In addition, when the worm extract was mixed with a recombinant RLuc, we did not detect luminescence using a luminometer. These results suggested that the luminescence system in the worm was independent of coelenterazine, although a RLuc homologous gene was found. Similarly, the existence of an RLuc homologous gene was reported in *P. atlanticum*, which has been suggested to use a coelenterazine-independent luminescence system relying on bacterial bioluminescent symbionts^30,35^. We also mixed the worm extract with a recombinant cypridinid luciferase, but we did not detect luminescence using a luminometer. This result was consistent with Harvey’s observation for *P. caliendrum*^16^. To examine whether the luminescence system is based on luciferin–luciferase reaction, which is found in various luminous animals including some syllid *Odontosyllis* spp*.*^23,36–39^, we prepared two different extracts of the worm using 100 mM HEPES–NaOH buffer (pH 7.4) and methanol, and subsequently subjected a mixture of the two to luminescent measurement. As a result, no light emission was detected from the mixture of the buffer and methanolic extracts of the worm. This result was also consistent with Harvey’s observation for *P. caliendrum*^16^. However, there is still a possibility that the light emission is based on luciferin–luciferase reaction, because luciferin–luciferase reaction found in fireflies or luminous mushrooms requires a cofactor such as ATP or NADPH, and we did not test all possible conditions due to the limitation of the number of collected specimens. In addition, extraction of luciferin and luciferase in the active form is sometimes difficult, as shown in previous studies^37^. Further studies using hundreds or more of the specimens must be performed to elucidate the mechanism underlying the violet-light emission.

## Methods

### Animal collection

The worms were collected in Ishikawa prefecture on Oct. 6–8, 2018 and on Oct. 13, 2019 and sorted under a microscope (SZ61; Olympus, Tokyo, Japan). For RNA extraction or UPLC analysis, a collected worm was put in a 1.5 mL tube with a sufficient amount of RNAlater (Thermo Fisher Scientific, Waltham, MA, USA) or approximately 500 μL of ethanol and stored with dry ice or at − 80 °C until use. Some of the fresh worms were put in a plastic tube with sea water and subjected to an electric stimulation of light emission.

### Materials

The commercially available materials used in this study were obtained from the following commercial suppliers. Coelenterazine was from FUJIFILM Wako Pure Chemical Corporation (Osaka, Japan). Coelenteramide and coelenteramine were from NanoLight Technologies, a division of Prolume Ltd. (Pinetop, AZ, USA). All materials were used without further purification. A recombinant *Renilla* luciferase was prepared using COSI cells and the pGL4.75 vector from Promega (Madison, WI, USA) according to the manufacturer’s protocol. A recombinant cypridinid luciferase from *Cypridina noctiluca* was prepared according to a method reported previously^40^.

### Equipment for photography and video recording

Photographs and videos were taken by a mirrorless camera (α7S; Sony, Tokyo, Japan) with a SEL24F18Z lens (Sony) and an underwater camera housing (Nauticam NA A7; Nauticam, Hong Kong, China).

### Measurement of luminescence emission spectrum

The luminescence emission spectrum of the worm *Polycirrus* sp. ISK stimulated by an electric shock was measured using a high-sensitivity charge-coupled device (CCD) spectrophotometer, LumiFLspectrocapture (AB-1850; ATTO, Tokyo, Japan) with the following settings: measurement mode, single; measurement time, 1 min; slit width, 0.25 mm; camera gain, high; diffraction grating, 150 lines/mm; and shutter for measurement, automatic. An anode and a cathode were put into the tube, and an electric pulse was generated using a 9 V battery.

### Luminescence monitoring of the worm stimulated by an aqueous solution of KCl

To a single specimen of the worm with 100 μL of natural sea water in a white 96-well plate (Nunc 96-Well polypropylene storage microplate; Thermo Fisher Scientific) was added 20 μL of a 4 M aqueous solution of KCl, followed by immediate measurement of luminescence intensity using a luminometer (Phelios AB-2350; ATTO) recorded in relative light units (RLU) in 0.02 s intervals over 30 s at room temperature.

### RNA-Seq and statistical analysis of differentially expressing genes

Using the RNeasy Plus Universal Mini Kit (Qiagen, Hilden, Germany), total RNA was extracted from the tentacles and the rest of body separated from a single specimen with dissecting instruments. cDNA libraries (100 bp pair-end) were prepared using the TruSeq standard mRNA sample prep kit (Illumina, San Diego, CA, USA) and sequenced by HiSeq 2500 (Illumina); 44.4 and 43.9 M reads yielded 4.49 and 4.43 Gbp for the tentacle and the rest of body samples, respectively. The raw reads were subjected to de novo assembly by using Trinity implemented in the MASER pipeline (National Institute of Genetics, NIG)^24^ available at http://cell-innovation.nig.ac.jp/maser/. After the assembly, sequence read mapping was performed using BWA-mem software^41^ implemented in the MASER pipeline (NIG)^24^, whereby the transcript expression levels were estimated to calculate the fragments per kilobase of exons per million (fpkm) values. Protein similarity was calculated using the blastP or blastX program (NCBI). A gene expression comparison between the tentacle and the rest of body was performed based on the ratio of TPM value and the subsequent WEGO analysis^27^. The DDBJ accession numbers for the RNA-Seq raw data and COI are DRR315406 and LC601006, respectively.

### Analysis of the worm extract using UPLC and mass spectrometry

The whole body of a single specimen stored in approximately 500 μL of ethanol at − 80 °C was homogenized in the storage ethanol on ice with a plastic pestle and centrifuged at 15,000×*g* for 5 min at 4 °C, after which 2 μL of the supernatant was subjected to luminescence analysis. A portion of the rest supernatant was five times diluted with 10% (v/v) acetonitrile in water and filtered through a centrifugal filter Ultrafree-MC (0.22 μm; Millipore, Billerica, MA, USA). Then, 10 μL of the filtrate was subjected to UPLC analysis and separation. UPLC analysis and separation were performed on a Waters ACQUITY UPLC H-Class system (Waters, Milford, MA, USA) equipped with an ACQUITY UPLC C18 column (ϕ2.1 × 100 mm, 1.7 μm; Waters) and an ACQUITY UPLC PDA eλ detector (Waters). The UPLC conditions were as follows: mobile phase, a linear gradient of acetonitrile in water from 10 to 100% for 20 min; flow rate, 0.3 mL/min; UV detection, 333 nm or 435 nm. The fraction eluted at a retention time of 9.5–10.0 min (panel a in Fig. 4A) was 20 times diluted with 1% formic acid and subjected to MS/MS analysis using an LCMS-9030 quadrupole time-of-flight mass spectrometer (Shimadzu, Kyoto, Japan). The parameters for MS/MS analysis were as follows: interface temperature, 300 °C; desolvation temperature, 250 °C; interface voltage, 4.5 kV; polarity, positive; collision energy, 30 V. Under the present UPLC condition, the detection limits were as follows: 15 pmol for coelenterazine, 0.1 pmol for coelenteramide, and 5 pmol for coelenteramine.

### Luminescence analysis of the worm extract

To 2 μL of the ethanolic extract of the worm in a white 96-well plate (Eppendorf microplate 96/F-PP; Eppendorf, Hamburg, Germany) was added 100 μL of a solution of a recombinant *Renilla* luciferase or cypridinid luciferase in 200 mM Tris–HCl (pH 7.5) containing 100 mM NaCl using the auto injector in the Phelios luminometer (ATTO), followed by the immediate measurement of luminescence intensity at room temperature. The concentrations of *Renilla* luciferase and cypridinid luciferase were sufficient to detect 100 fmol of coelenterazine and 100 amol of cypridinid luciferin, respectively.

## Supplementary Information

Supplementary Information 1.

Supplementary Information 2.

Supplementary Information 3.

Supplementary Video S1.

Supplementary Video S2.

Supplementary Video S3.
