# Supplementary material for: Violet bioluminescent Polycirrus sp. (Annelida: Terebelliformia) discovered in the shallow coastal waters of the Noto Peninsula in Japan
Source: Sci Rep. 2021 Sep 27;11:19097. doi: 10.1038/s41598-021-98105-6 (PMC8476577; doi:10.1038/s41598-021-98105-6)
Supplement: Supplementary file 1 — Supplementary Information 1. [file 41598_2021_98105_MOESM1_ESM.pdf]

Supplementary information

Violet bioluminescent *Polycirrus* sp. (Annelida: Terebelliformia) discovered in the shallow coastal waters of the Noto Peninsula in Japan

Shusei Kanie[1], Daisuke Miura[2], Naoto Jimi[3], Taro Hayashi[4], Koji Nakamura[5], Masahiko Sakata[5], Katsunori Ogoh[4], Yoshihiro Ohmiya[6, 7], Yasuo Mitani[1,*]

[1]Bioproduction Research Institute, National Institute of Advanced Industrial Science and Technology (AIST), Sapporo 062-8517, Japan; [2] Biomedical Research Institute, AIST, Tsukuba 305-8566, Japan; [3]National Institute of Polar Research, Tachikawa, Tokyo 190-8518, Japan; [4]Olympus Corporation, Hachioji, Tokyo 192-8507, Japan; [5]Japan Underwater Films Co., Ltd., 2-11-15, Nakaochiai, Shinjyuku, Tokyo 161-0032, Japan; [6]Biomedical Research Institute, AIST, Ikeda 563-8577, Japan; [7]Osaka Institute of Technology (OIT), Osaka 535-8585, Japan

*Corresponding author e-mail: mitani-y@aist.go.jp

Index

1. Supplementary figures **page S3**
2. Supplementary tables **page S9**
3. Legends for supplementary videos **page S11**
4. Supplementary references **page S12**

Supplementary figures

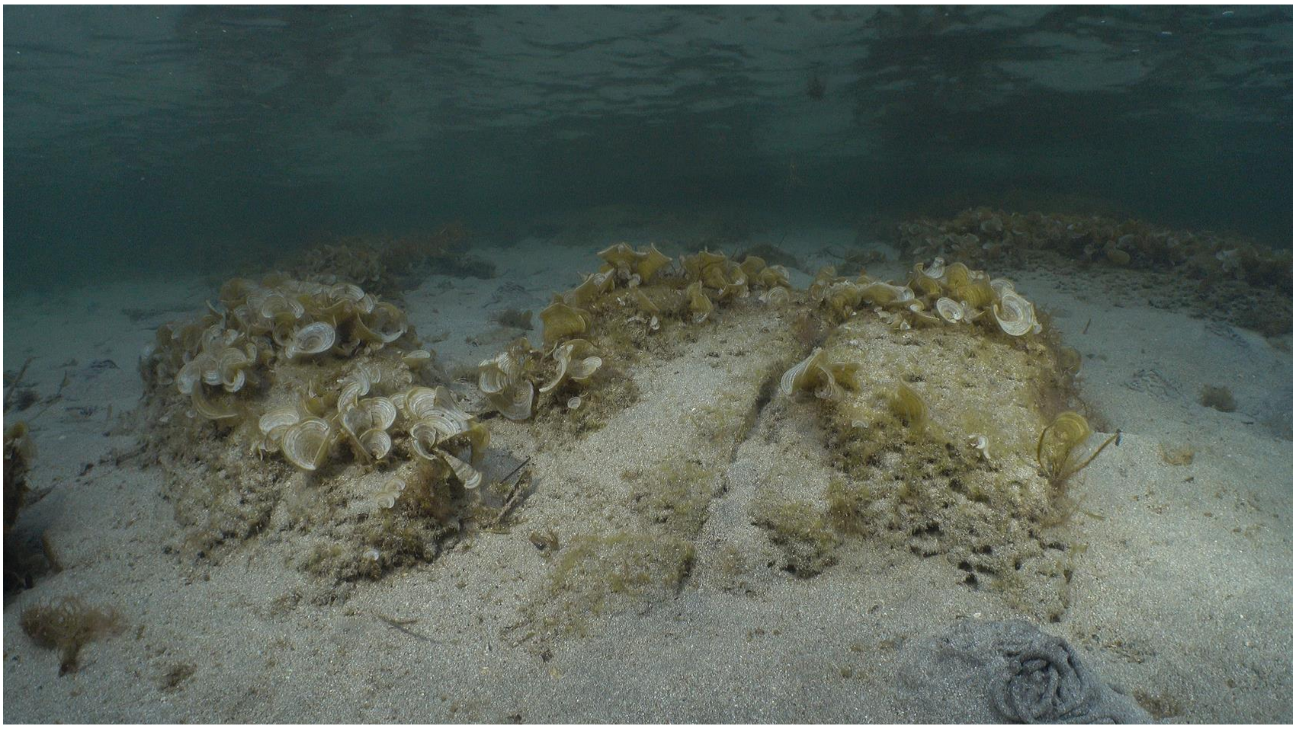

Supplementary Figure S1. Photograph in the living environment of *Polycirrus* sp. ISK.

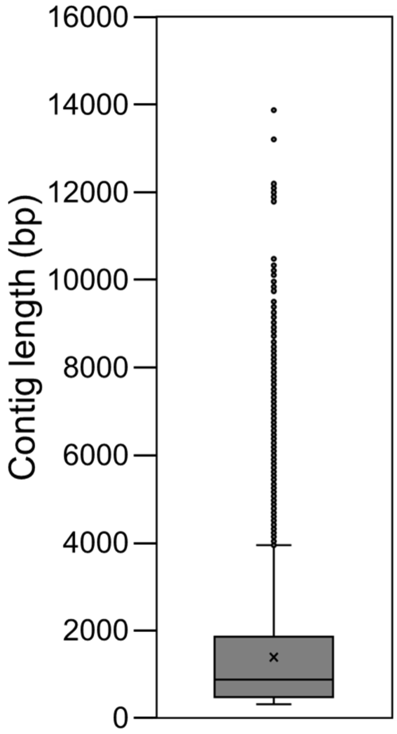

Supplementary Figure S2. Box plot diagram of the sizes of transcripts obtained by RNA-Seq analysis.

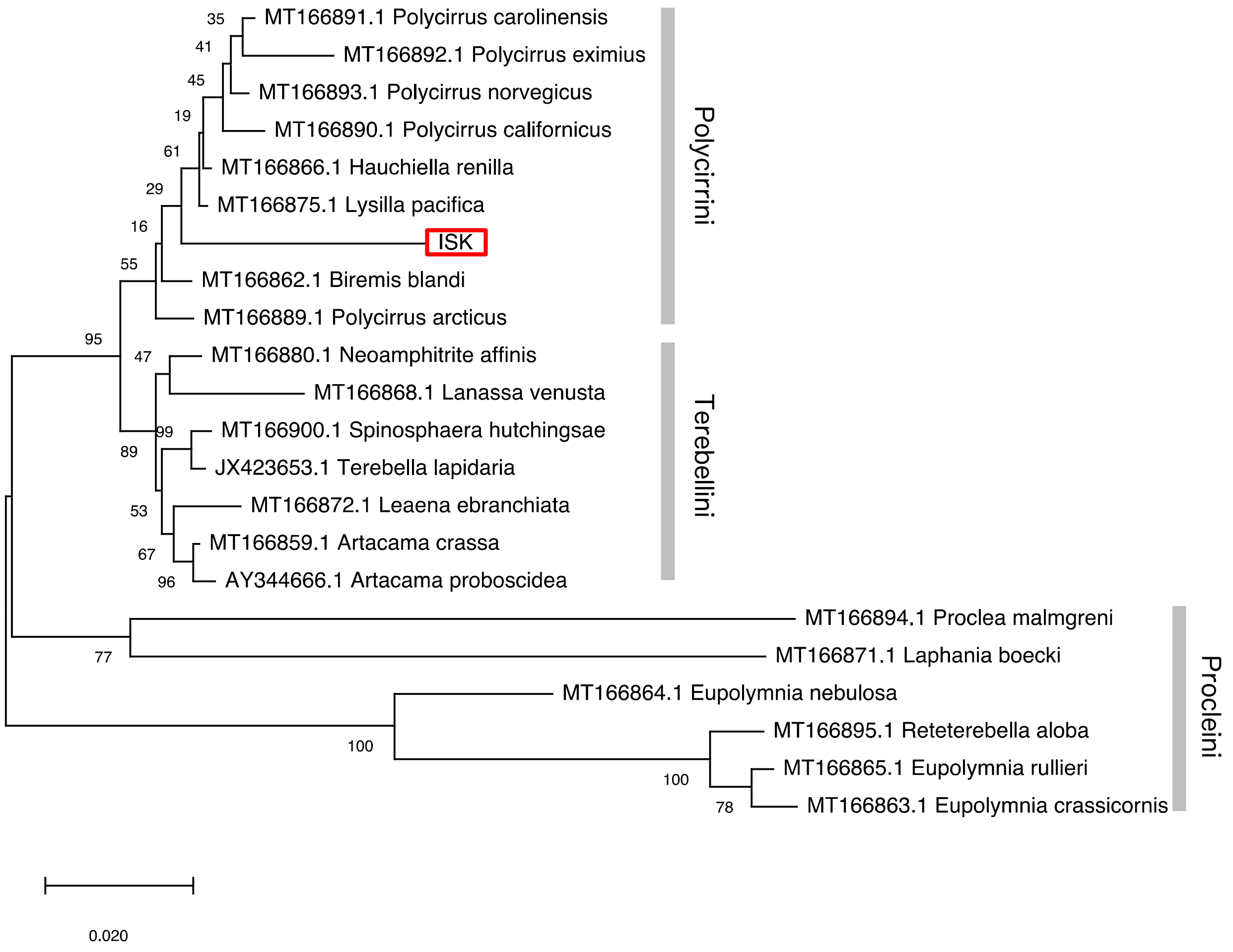

Name	Sequence
COI_aa	LYSTNHKDIGTLYFIFGVWGGLLGTSMSLLIRIELGQPGAFLGSDQLYNTIVTAHGLLMIFFLVMPI<br>LIGGFGNWLVPLMLGAPDMAFPRMNNMSFWLLPPALLLLLSSAAVEKGAGTGWTVYPPLASNLAHAG<br>PSVDLAIFSLHLAGISSILGAINFITTVANMRWKGLRLERIPLFVWAVNITVILLLLSLPVLAGAIT<br>MLLTDRNVNTSFFDPSGGGDPILYQHLFWFFGHPEVYILILPGFGAISHIVTHYSGKLEPFGTLGMI<br>YAMLGIGILGFIVWAHHMFTVGMDVDTRAYFTAATMIIAVPTGIKVFSWLATIFGSRIKYETPMLWA<br>LGFIFLFTMGGLTGIVLANASLDAALHDTYYVTAHFHYVLSMGAIFAIFAGFNHWFPLFAGITLHYR<br>WAKIHFIMMFIGVNLTFFPQHFLGLSGMPRRYSDYPDAYTKWNVVSSFGSMISFISLIFFIFILWEA<br>FSAQRPIIGSTHMPTSLEWQDILPLDFHNLPETGIITKL

Supplementary Figure S3. Molecular phylogenetic analysis of COI and its amino acid sequence. To construct the molecular phylogeny tree, deduced amino-acid sequences were aligned using the MUSCLE algorithm implemented in MEGA X version[1] available at https://www.megasoftware.net. Molecular phylogenetic analyses were conducted by the maximum-likelihood method, and the molecular phylogenetic tree was generated using MEGA X version[1]. Bootstrap values for the maximum likelihood phylogenies were obtained by 1,000 replications.

```
ISK               --------LILCQNLYYYICTNIIHFLSDSAGAWTALSEVFPTVTMQG------------
O_undec           MKLALLLSIGCCLVAVNFALRATIIRCLRKTRSWSEIDCTPHQDKLYEDFDRIWAGDYLS
                          :  *     :     *     .: :*: :. .    .:

ISK               CVFHWVQS----VWS--KVQNVGLQATYNQRQDVYDYISKLMALPFLPSKHIRPAFET--
O_undec           VFAEWLDNPIPREWSEERLATYCIERECHTNQAMVDYMNIHGYAPFCMERSVEDWVNARF
                   . .*::.     **  :: .  ::   : .* : **:.     **  .: :. .::

ISK               -LKAKAHTEPLQQLVQYMERTWFSSTVFTMQS--------------------ICMHNKFI
O_undec           WTRCKVRTDRSLELAPEEYATYFCYKVFRVQDPKIACPSMDVILSPNKLTVQQMMQNKEI
                    :.*.:*:   :*.     *:*. .** :*.                      *:** *

ISK               R--TNNDVEGWHRRINKRASGQSLGFYVLVPFLLKEALFVDTQLRLVQDG----------
O_undec           RGVVEDRSEQWWVGLMREISHLSKDLNGVKQFHYGWIINTATQKNVVPLWSRYQGPTIPV
                  *  .::  * *   : :. *  * .:  :  *     : . ** .:*

ISK               -------------------------------------LLSSDNSLSYAQAQDSLMDLWR-
O_undec           RRDMPRIINAMSNGGGNITLGDIRNFHCSPDPDSVAVICPEFGFLSYSPAETIVMVPVNG
                                                       : .. . ***: *:  :*   .

ISK               RYDAKLIQTADFLSQAAESYGQF------
O_undec           LILMGMTQSTDGVPFVKSALFAEMYNLQQ
                       : *::* :. . .:
```

Supplementary Figure S4. Sequence alignment of putative protein sequence that exhibits similarity to *Odontosyllis undecimdonta* luciferase sequence with an e-values of more than $1e^{-10}$. ISK, *Polycirrus* sp. ISK; O_undec, *Odontosyllis undecimdonta*. The alignment was performed using ClustalW (DNA Data Bank of Japan)[2]. Asterisks (*) indicate identical amino acid residues. Colons (:) indicate sites belonging to group exhibiting strong similarity. Periods (.) indicate sites belonging to a group exhibiting weak similarity. The site similarity followed the criteria of ClustalW.

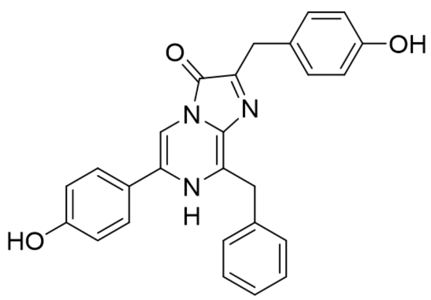

Coelenterazine
(CTZ)

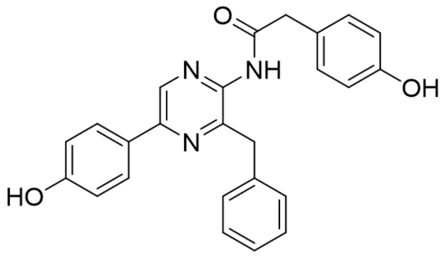

Coelenteramide
(CTMD)

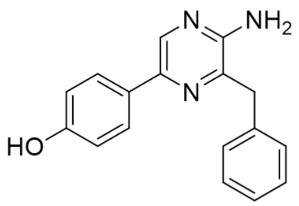

Coelenteramine
(CTM)

Supplementary Figure S5. Chemical structures of CTZ, CTMD, and CTM.

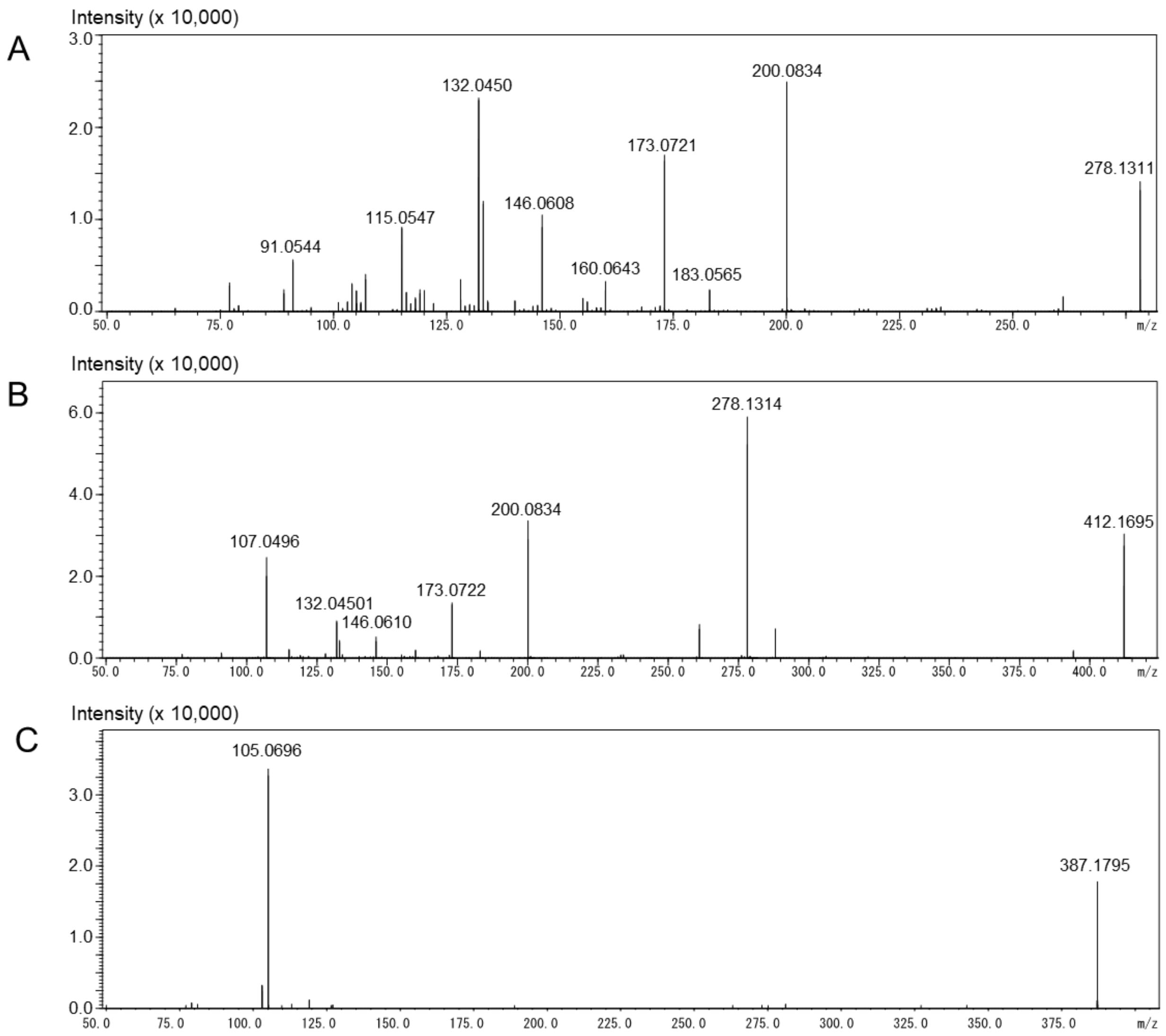

Supplementary Figure S6. Comparison of MS/MS spectra of the compound obtained by UPLC separation and authentic standards. (A) authentic CTM, (B) authentic CTMD, (C) compound between the red vertical dashed lines shown in panel (a) in Figure 4A.

Supplementary tables

Supplementary Table S1. Top 8 tentacle-specific transcripts selected on the basis of fpkm values.

Target ID	length	fpkm/tentacle (TPM/tentacle)	fpkm/body (TPM/body)	homology	score	e-value	domain
comp14334_c0_seq1	330	1603.35 (537.33)	0.53 (0.18)	None	-	-	-
comp11925_c0_seq1	874	1125.74 (826.04)	0.82 (0.62)	CD97 antigen-like [*Dendronephthya gigantea*]	67.4	2e-08	C-type lectin (CTL)/C-type lectin-like (CTLD) domain
comp10434_c0_seq1	766	1642.18 (976.67)	2.08 (1.10)	macrophage mannose receptor 1-like isoform X2 [*Leptotrombidium deliense*]	47.4	0.037	C-type lectin-like domain (CTLD) of the type found in the type 1 transmembrane proteins
comp40876_c1_seq6	814	4084.85 (2836.46)	12.47 (10.35)	dermatopontin [*Suberites domuncula*]	129	7e-33	Dermatopontin; Members of this family mediate cell adhesion via cell surface integrin binding. ...
comp36676_c0_seq2	1360	1268.60 (1682.73)	4.43 (5.84)	ricin-type beta-trefoil lectin domain protein [unclassified *Xanthobacter*]	84.3	4e-15	Ricin-type beta-trefoil lectin domain
comp28076_c0_seq1	1317	1281.03 (1077.27)	4.90 (4.25)	ricin-type beta-trefoil lectin domain protein [*Leptolyngbyaceae cyanobacterium* SL_5_14]	97.8	3e-19	Ricin-type beta-trefoil; Carbohydrate-binding domain formed from presumed gene triplication. ...
comp33211_c0_seq2	855	1757.16 (1343.02)	8.55 (6.90)	None	-	-	-
comp42157_c1_seq1	1179	1151.38 (778.64)	5.70 (3.62)	None	-	-	-

Supplementary Table S2. Top 8 body-specific transcripts selected on the basis of fpkm values.

Target ID	length	fpkm/tentacle (TPM/tentacle)	fpkm/body (TPM/body)	homology	score	e-value	domain
comp17641 _c0_seq1	496	0 (0)	5086.48 (2922.66)	None	-	-	-
comp28611 _c0_seq1	558	0 (0)	5046.03 (3151.77)	None	-	-	-
comp23953 _c0_seq1	564	0 (0)	3610.10 (2068.62)	None	-	-	-
comp33221 _c0_seq5	512	0 (0)	3607.03 (2495.98)	None	-	-	-
comp42552 _c0_seq1	276	0 (0)	2135.55 (977.18)	None	-	-	-
comp41241 _c0_seq5	474	0 (0)	2098.06 (1175.04)	None	-	-	-
comp25545 _c0_seq2	484	0 (0)	2084.58 (1061.89)	None	-	-	-
comp33871 _c0_seq1	883	0 (0)	1035.92 (845.19)	hypothetical protein CAPTEDRAFT_117854, partial [*Capitella teleta*]	152	1e-41	None

Legends for supplementary videos

Supplementary Video S1. Luminous behavior of *Polycirrus* sp. ISK in the surface of the rock at the shallow sea bottom. The video was recorded with the following settings: sensitivity, ISO 51200; white balance, 4300 K; shutter speed, 1/30 s; iris, F3.5; frame rate, 29.97 fps; frame size, 1920 × 1080 pixels.

Supplementary Video S2. Luminous behavior of *Polycirrus* sp. ISK in the rock at the shallow sea bottom. The video was recorded with the following settings: sensitivity, ISO 51200; white balance, 4300 K; shutter speed, 1/30 s; iris, F3.5; frame rate, 29.97 fps; frame size, 1920 × 1080 pixels.

Supplementary Video S3. Luminous behavior of *Polycirrus* sp. ISK in our laboratory condition. This worm was stimulated by an electric shock in our laboratory. The video was recorded with the following settings: sensitivity, 11 lux; white balance, 5800 K; shutter speed, 1/60 s; iris, auto; frame rate, 60 i; frame size, 1920 × 1080 pixels.

Supplementary references

1. Kumar, S., Stecher, G., Li, M., Knyaz, C., Tamura, K. MEGA X: Molecular evolutionary genetics analysis across computing platforms. *Mol. Biol. Evol.* **35,** 1547–1549 (2018).
2. ClustalW. Available at: https://clustalw.ddbj.nig.ac.jp/ (accessed on 7 August 2021).
